# Investigating the Yanomami malaria outbreak: gold mining and malaria

**DOI:** 10.1098/rsbl.2025.0659

**Published:** 2026-01-07

**Authors:** Daniela de Angeli Dutra, Jesus Fontes, Érika Martins Braga, Erin Mordecai

**Affiliations:** 1 Department of Biology, Stanford University, Stanford, CA, USA; 2 School of Environmental and Natural Sciences, Bangor University, Bangor, UK; 3 Faculdade de Medicina, Universidade Federal de Mato Grosso, Cuiabá, Brazil; 4 Departmento de Parasitologia, Universidade Federal de Minas Gerais, Belo Horizonte, Minas Gerais, Brazil

**Keywords:** malaria, Amazonia, Brazil, gold mining, forest loss, land use, Yanomami, Pathogen biology

## Abstract

The Yanomami, an Indigenous group from the Amazon, confront multifaceted challenges endangering their health and cultural integrity. Of immediate concern is the humanitarian crisis caused by surges in malaria amid increasing illegal gold mining in their territory. Leveraging satellite imagery and panel regression analyses, we quantified the effect of land use changes on malaria incidence on their land (2016–2023). We observed an approximately 300% increase in malaria cases during this period, associated with increases in illegal gold mining. An increase of 1 s.d. in gold mining is associated with a 20–46% rise in malaria incidence 1–2 years later. We found that changes in forest areas significantly affect malaria rates: for every 1 s.d. increase in the perimeter of forest edges, malaria cases rise by 55%. Our findings highlight the major impact of illegal gold mining and the resulting fragmentation of forests on the high malaria burden experienced by the Yanomami.

## 1. Introduction

Human malaria is a vector-borne disease caused by *Plasmodium* spp. parasites, transmitted by around 70 species of anopheline mosquitoes. It remains one of the leading causes of infectious disease globally, with an estimated 263 million cases and 597 000 deaths in 2023 [1]. Malaria is concentrated in sub-Saharan Africa and tropical regions of Asia, Oceania and Latin America [1]. In Brazil, malaria cases declined substantially from 500 000 to 600 000 cases annually in the 1990s to 100 000 to 200 000 cases per year in the 2000s, due to intensified control measures [2]. Subsequently, malaria has increased by 300% since 2000 in Indigenous territories and regions subject to illegal gold mining (i.e. ‘garimpo’) [3]. Deforestation also increases malaria incidence in Brazil [4,5], demonstrating that changes in land cover alter the local risk of malaria. During the presidential administrations of Michel Temer and Jair Bolsonaro (from 2016 to 2022), deforestation and illegal gold mining increased in Brazil [6,7]. In particular, Bolsonaro’s administration weakened environmental and Indigenous protections, encouraging illegal mining, logging and agriculture in the Amazon by transferring Indigenous territory demarcation to the agriculture ministry and encouraging illegal gold mining through rhetoric and policy neglect [8–10]. This led to a 625% increase in illegal gold mining in Indigenous territories from 2013 to 2022 [11], along with reduced health services and increased threats to Indigenous land rights [12]. These changes could be affecting the recent malaria outbreaks in Indigenous communities, including the Yanomami in northern Brazil.

The Yanomami are an Indigenous group of 35 000–40 000 people, of whom approximately 30 000 live in Brazil according to the Brazilian Indigenous Health Secretary (SESAI) database (‘População Indígena’). Recently, the Yanomami experienced a humanitarian crisis triggered by the invasion of 20 000 illegal miners into their territory, which was internationally recognized in January 2023 [13,14]. This humanitarian crisis was characterized by severe malnutrition, poor access to healthcare, mercury contamination due to illegal gold mining and high malaria burden [9,10]. In particular, the malaria outbreaks during this period may have been caused by surges in illegal gold mining in the region. Anthropogenic land use change can increase habitat for the vector, *Nyssorhynchus darlingi*, which thrives in forest edges and disturbed forests [15]. Illegal gold mining drives malaria transmission through four mechanisms: creating water pools for vector breeding, facilitating pathogen spread through miner movement, delaying malaria treatment in miners leading to increased local transmission, and contaminating waterways with mercury, which weakens immune responses and increases malaria susceptibility (electronic supplementary material, figure S1) [16–18]. Although media reports and scientific articles have discussed these threats to the Yanomami [13,17,19], there has not yet been a formal analysis to quantify the impacts of illegal gold mining and other land use changes on the malaria outbreaks in the Yanomami land during this crisis period.

Brazilian National Plan for Malaria Elimination (Plano Nacional de Eliminação da Malária), which is based on timely diagnoses and treatment, aims to eliminate malaria deaths by 2030 and cases by 2035 [20]. While Indigenous lands and mining sites faced surges in malaria in the last 20 years, most of the country succeeded in reducing malaria transmission. For Brazil to eliminate malaria, one necessary action is to uncover the impact of illegal gold mining on malaria incidence, particularly in Indigenous lands. For this reason, we evaluated and quantified whether illegal gold mining and other land use changes drive malaria incidence on the Yanomami land. We considered both immediate and 1 and 2 years lagged impacts of illegal gold mining on malaria. We show that illegal gold mining and increases in forest edge perimeter are the main changes in land use linked to increases in malaria among the Yanomami.

## 2. Methods

### (a) Data

**Figure 1.**
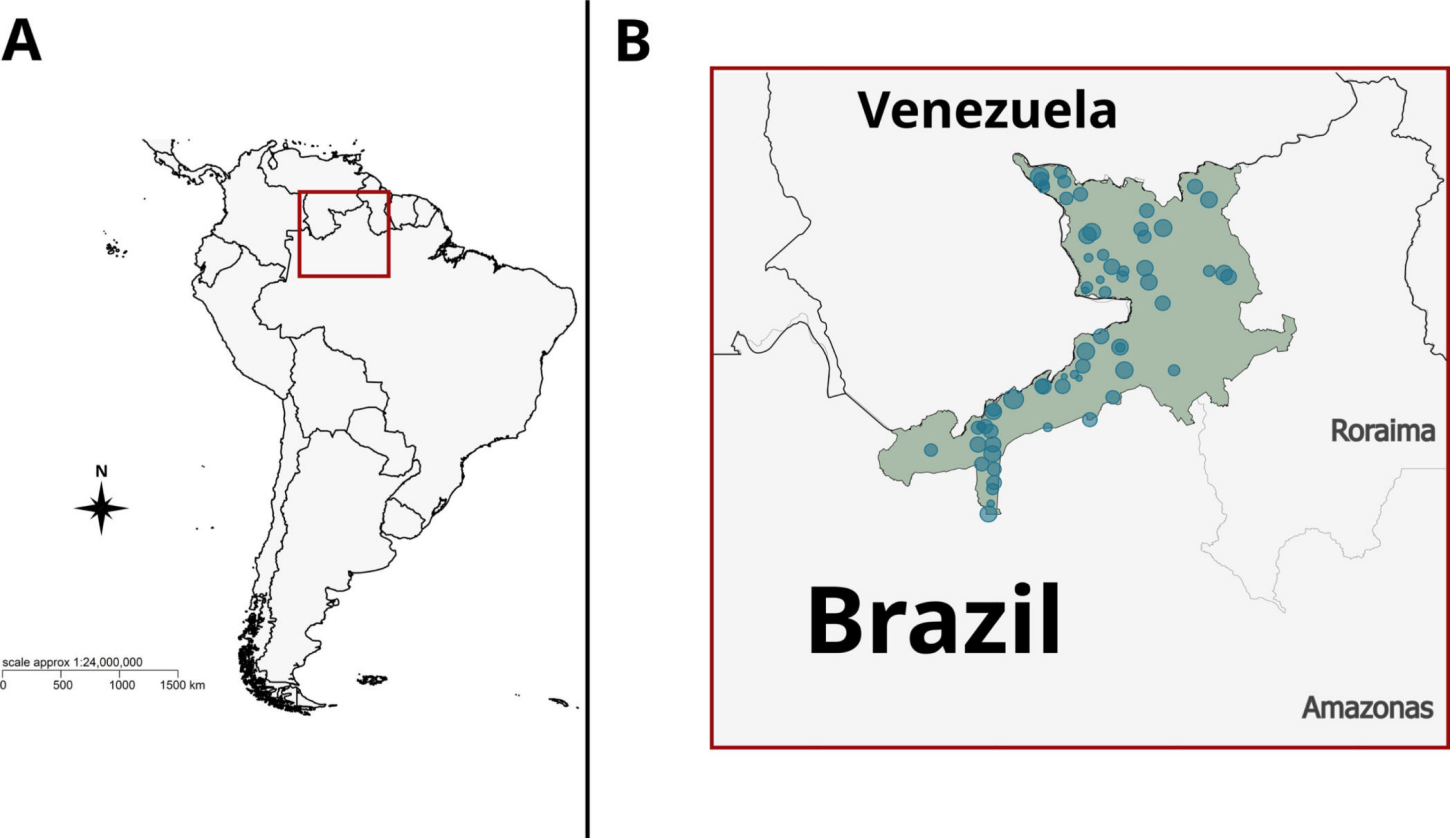
Yanomami Indigenous land and infection sites. (A) Location of the Yanomami territory in South America. Red square represents the area highlighted in (B). Yanomami Indigenous land (in green) and infection sites (our spatial units, in blue). Circle diameters depict the total log2 number of malaria cases in each Yanomami infection site. Borders in Brazil represent the states of Roraima and Amazonas.

In December 2023, we obtained annual data on malaria cases in Brazil between January 2003 and September 2023 from the Brazilian Ministry of Health database provided by the Indigenous Special Secretary of Health (SIVEP-SESAI Malaria, Malaria em áreas indígenas table) and filtered the data to include only cases reported at the Yanomami Indigenous Sanitary Special District. Malaria data in the Yanomami territory is reported at the polo base level (i.e. health district subdivisions) and typically associated with a coordinate point indicating the likely site of infection (hereafter, ‘infection sites’) (figure 1). We selected the variables: year, infection site (i.e. coordinate point location associated with diagnosed malaria cases), parasite species, population and polo base. In total, 28 polo bases reported malaria in 64 distinct infection sites. We filtered the malaria data (approx. 480 000 malaria records) to only include cases among the Yanomami people (approx. 120 000 malaria records). The population size at each polo base (i.e. Indigenous health division), dated from 2010 to 2023, was used to calculate malaria incidence. Although this approach might deflate incidence in polo bases containing multiple infection sites, it allows assessment of changes in incidence over time while maintaining the highest resolution on focal land use change, which is the primary aim of this research. For the years before 2010, estimates from 2010 were used to calculate the incidence. Forest and mining cover data were obtained through the Google Earth Engine platform [21] using the MapBiomas Brazil 9.0 database (https://brasil.mapbiomas.org). Climate data (i.e. annual mean temperature and total precipitation) was obtained from the Climate Research Unit (CRU) database [22]. See details on spatial analyses in the supplementary methods. Our data, along with coding, is openly available from the Dryad Digital Respository [23].

### (b) Statistical analyses

Because of the robustness of panel regression models in identifying and quantifying ecological relationships [24], we used two-way fixed-effects panel regression models to assess the effects of land use change and illegal gold mining on malaria incidence among the Yanomami. Our model accounts for both observed and unobserved variation across sites and years by incorporating spatial unit and year fixed effects as well as climate covariates, thereby reducing the potential impact of confounding variables that could bias the estimated relationship between land use change and malaria incidence. This occurs because fixed effects effectively address omitted variable bias, encompassing both observed and unobserved confounders [24], including shared time shocks, such as large-scale climatic events and political administration changes, as well as temporally constant spatial variation, like topography and geography. Rather than including unit-level (i.e. infection site) spatial fixed effects, which resulted in model overfitting, we aggregated the spatial fixed effects to the polo base level.

For all statistical analyses, we used the package ‘fixest’ [25] in R version 4.3 [26]. We ran a two-way fixed-effects panel regression model to evaluate the effects of land use and climate variables on the incidence of malaria cases in the Yanomami people across the 64 infection sites. The panel data have a hierarchical structure, with units of analysis being each polo base in each year. This ensures that our estimates of marginal effects of the explanatory variables of interest are not confounded by cross-sectional or shared temporal variation. We consider the incidence of malaria per site and year (i.e. the total number of malaria cases divided by population for each year and infection site) as our response variable. Scaled annual forest cover, change in illegal gold mining cover, forest growth, forest edge perimeter, mean temperature and total precipitation were used as explanatory variables in our model. The model used total malaria incidence as the response variable. Because malaria incidence data are non-negative and right-skewed, we used a Poisson panel regression model with a log link. The formula describing the model is as follows:

(2.1) $$log(E[Yit])=\lambda_{i}+\mu_{t}+\text{Θ}{\text{X}}_{it}+\varepsilon_{it},$$

where

- E[Yit] is the expected value (mean) of malaria incidence in each polo base i and t year, which is linked to the predictors through a logarithmic (log) function in the Poisson model.
- $\lambda_{i}$ and $\mu_{t}$ are the fixed effects associated with each spatial and temporal unit (i.e. polo base i and year t).
- *i* and *t* represent the indices for infection sites (units) and years, respectively.
- Θ are the coefficients associated with the control variables ${\text{X}}_{it}$ (i.e. forest cover, change in illegal gold mining cover, secondary forest growth, forest edge perimeter, mean temperature and total precipitation).
- $\varepsilon_{it}$ represents the error term.

Since we used Poisson log-link models, we exponentiated the coefficients to calculate incidence rate ratio $\left(\text{IRR=}e^{\theta }\right)$. All analyses were repeated using only *Plasmodium falciparum* incidence (the parasite species associated with higher morbidity) as the response variable and, for both total and *P. falciparum* malaria, with 1 and 2 years lagged mining changes as explanatory variables. Lagged mining models were run to distinguish the short-term (e.g. increased mobility and mortality) and long-term (e.g. mercury contamination) impacts of illegal gold mining on malaria incidence. All models were weighted by population size in each infection site in each year without controlling for human movement. Details of spatial autocorrelation and robustness checks, models and limitations of our study can be found in electronic supplementary material, methods.

## 3. Results

### (a) Temporal trends in malaria cases

**Figure 2.**
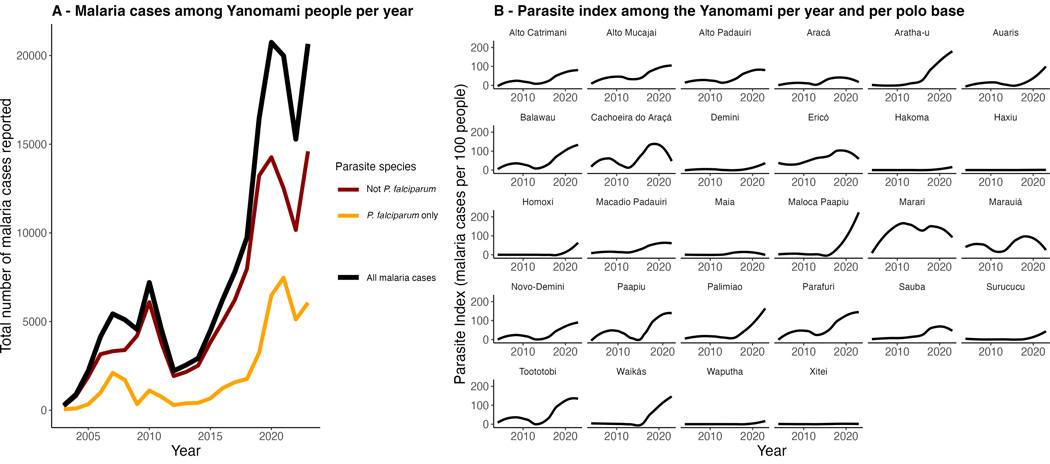
Trend over time of the total number of malaria cases. (A) All malaria cases (black line), malaria cases excluding *P. falciparum* (maroon line), and *P. falciparum* only malaria cases (gold line) reported among the Yanomami from 2003 to 2023. (B) Parasite index (malaria cases [all parasite species] per 100 people) among the Yanomami in each Indigenous polo base from 2003 to 2023. Malaria cases for the 64 infection sites were aggregated into their respective polo bases.

Malaria cases in the Yanomami increased by 300% after illegal gold mining increased in their territory, from 2016 to 2023, to a high of over 20 000 annual cases in 2022 (figure 2A). Previously, malaria had reached a low of 276 cases in 2003, increased to 7204 in 2010, decreased again from 2011 to 2014, then began to increase in 2015 (figure 2A). When plotting malaria dynamics by polo bases (health subdivisions) encompassing multiple infection sites (i.e. the spatial unit of our model), we observe that incidence trends were not spatially homogeneous; for instance, no change in malaria parasite index (i.e. cases per 100 people) over time was observed in certain polo bases (figure 2B).

### (b) Changes in land use in the Yanomami Indigenous land

**Figure 3.**
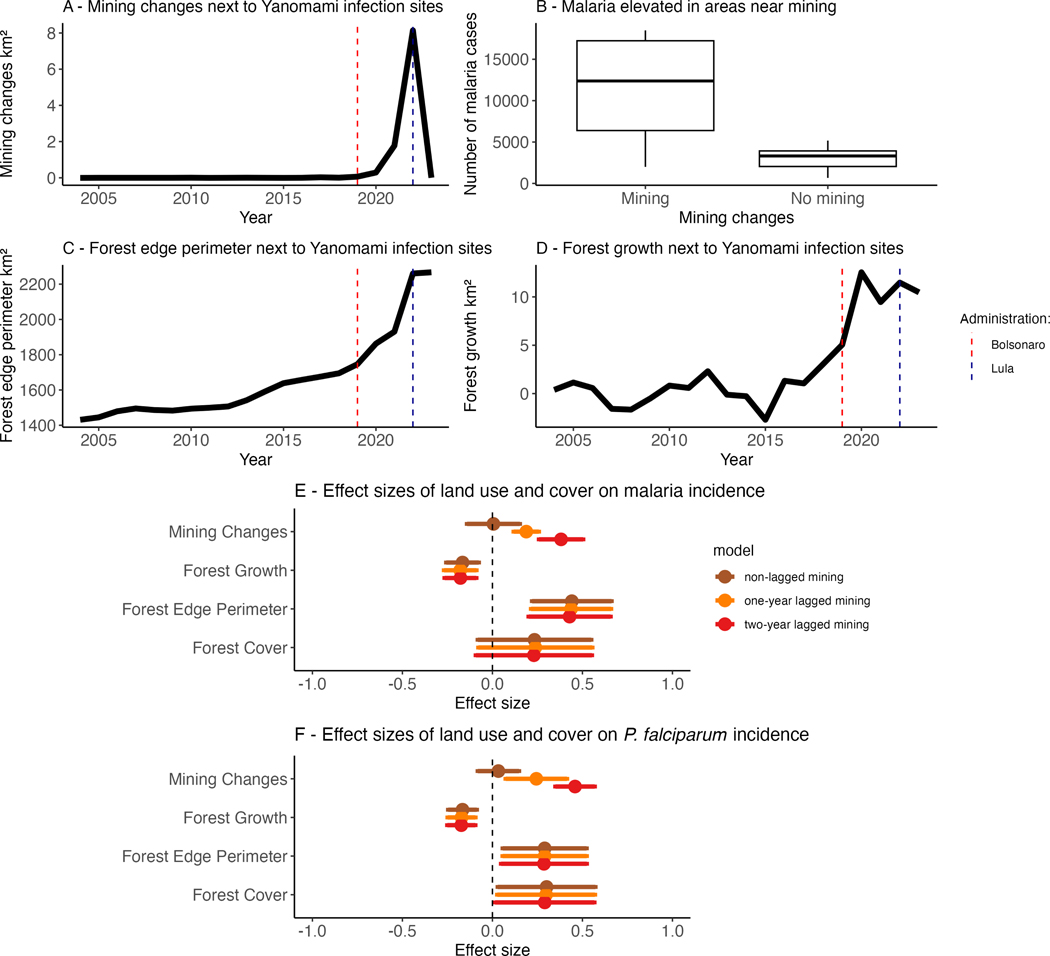
Land use change in Yanomami territory from 2003 to 2023. (A) Temporal variation in mining cover next to Yanomami infection sites (10 km radius). (B) Boxplot indicating the median (horizontal line) and interquartile range (box) number of malaria cases reported per unit time in infection sites and periods with and without mining activity. (C) Temporal variation in forest edge perimeter next to Yanomami infection sites (10 km radius). (D) Temporal variation in forest growth next to Yanomami infection sites (10 km radius). (E,F) Effect sizes of land use and cover on malaria (all cases) and *P. falciparum* incidence in non-lagged and 1 and 2 years lagged mining changes models (brown, orange and red points, respectively). Error bars represent coefficient intervals; effects are considered statistically significant when these intervals do not overlap zero. Dashed red lines mark Bolsonaro’s term; dashed blue lines mark Lula’s.

We observed increased illegal gold mining cover next to the Yanomami infection sites (i.e. coordinate points associated with diagnosed malaria cases) starting in 2020 (during Bolsonaro’s administration), increasing until 2022 (figure 3A). This was followed by a sharp decrease in mining activities in 2023 (figure 3A), during Lula’s administration. Within the entire Yanomami Indigenous territory, increases in mining started in 2016 and continued through 2022, surging from approximately 0.04 to 32 km^²^. An increase in secondary vegetation growth and forest edge perimeter occurred next to the Yanomami infection sites from 2019 to 2023 (figure 3C,D). The fact that the surge in mining in the Yanomami territory coincided in time (figure 3A) and space (figure 3B) with the largest numbers of malaria cases suggests that illegal gold mining contributed to the malaria burden suffered by the Yanomami: a hypothesis we tested with a statistical model.

### (c) Drivers of malaria in the Yanomami territory

To investigate the drivers of this surge in malaria cases among the Yanomami people, we ran two-way fixed-effects panel regression models to quantify the effect of land use change (e.g. illegal gold mining) on the incidence of malaria (all parasite species) and *P. falciparum* malaria. Our models point to illegal gold mining as a main driver of malaria cases in Yanomami territory, with every 1 s.d. (0.03% cover change) increase in illegal gold mining linked to increases in malaria incidence of 20 and 46% (estimates = 0.188 and 0.381) in the 1-year lagged and 2-years lagged models, respectively (table 1, figure 3E). No statistical association between mining and malaria was found in non-lagged models. Similar effect sizes of gold mining on *P. falciparum* malaria incidence were estimated using the mining lagged models (table 2). Illegal gold mining showed consistent associations with malaria incidence across all lagged models, except those excluding outliers (electronic supplementary material, tables S1–S12).

Forest growth and edge perimeter also affected malaria. Changes in forest edge perimeter increase malaria incidence among the Yanomami, with every 1 s.d. increase in forest edge (5.26% change) linked to a 55% increase in malaria incidence (table 1), with smaller effects (33%) for *P. falciparum* incidence (table 2). Forest edge perimeter was positively associated with malaria in all models, while forest cover was positively associated only in models with non-lagged gold mining (electronic supplementary material, tables S1–S12). Forest growth (i.e. positive change in forest cover from the preceding year) had a protective effect against malaria, with a 1 s.d. increase in forest growth linked to a 15.3% decrease in malaria incidence (tables 1 and 2). This effect was consistent across all models (electronic supplementary material, tables S1–S12). We observed no consistent association between climate and malaria incidence (tables 1 and 2, electronic supplementary material, tables S1–S12), potentially because these varied modestly at the spatial scale of the analysis and year fixed effects absorb interannual climatic variation that is shared throughout the territory. More details from our robustness check models can be found on electronic supplementary material.

## 4. Discussion

### (a) Gold mining and malaria in the Yanomami territory

Vector-borne disease incidence is sensitive to multiple socio-environmental drivers, including land use, climate, population and human behaviour [4,27–29]. Here, we quantify the impact of illegal gold mining on malaria incidence in the Yanomami territory: every 1 s.d. increase in illegal gold mining is linked to 20–40% increases the incidence of malaria 1–2 years later. Those are possibly linked to lagged effects of illegal gold mining (e.g. mercury contamination). The increase in illegal gold mining activities in the Yanomami territory started in 2016 and surged from approximately 0.04 to 32 km²; since then, malaria cases have increased from less than 5000 to approximately 20 000 malaria cases (figure 2A). The fact that the forest edge perimeter increases coincided with both increases in illegal gold mining and forest growth suggests that these are likely linked to illegal gold mining. Forest edge expansion had the largest scaled effect on malaria incidence—55% increase for each standard deviation increase in forest edge—of any land use change we examined. This suggests that another mechanism connecting illegal gold mining to malaria is the creation of vector breeding habitats, which are primarily located at forest edges. Significant estimates persist even when considering only *P. falciparum* malaria (tables 1 and 2). Increases in *P. falciparum* cases are particularly concerning as those are an indicator of reductions in malaria control [2]. This is because *P. falciparum* gametocytes typically appear 7–15 days after the onset of symptoms, indicating a lack of timely diagnosis and treatment. Since the analyses cannot distinguish among causal mechanisms and potential confounders that covary with land use change in space and time, field work directly investigating changes in vector habitat, parasite importation, mobility and mercury exposure is necessary to more definitively evaluate these hypotheses.

Trends of increased mining in Indigenous territories from 2019 onward support the idea that Bolsonaro’s anti-environmental policies contributed to the explosion of illegal gold mining and environmental degradation throughout the country [10,30], with over 20 000 illegal miners occupying the Yanomami territory in January 2023 [31]. Another major external driver of these dynamics is the concurrent increase in international gold prices [32], which have been associated with increases in gold mining activities and malaria incidence in multiple countries in the Global South [32–34]. Therefore, surges in illegal gold mining and malaria in the Yanomami territory are likely also driven by the increasing profitability of the gold market in the last few years, abetted by lax environmental policies and protections for Indigenous people and land.

### (b) Gold mining and malaria control

Gold mining is associated with surges in malaria cases in many countries in Africa (e.g. Ghana and Nigeria), Asia (e.g. Thailand, Cambodia and Myanmar), and South America (e.g. Brazil, Colombia and Peru) [32–35]. These surges pose an obstacle to malaria elimination and control programmes as miners are frequently non-immune itinerant workers who often become infected and do not receive adequate treatment [33]. This leads to the persistence of infectious hosts in mining settlements and boosts pathogen resistance to anti-malarial treatment through improper usage [33]. In the Amazon, gold mining-driven malaria transmission is spatially heterogeneous, with most cases being reported in health centres distant from main roads and close to illegal gold mining sites [34]. External factors, including the immigration of people from malaria hotspots in Venezuela, likely affected the transmission of malaria in the Amazon. For example, the rise in *P. falciparum* cases in northern Brazil (2016–2020) was driven by Venezuelan migration, especially among miners [36]. Despite the fact that Brazilian malaria elimination programmes have significantly reduced malaria in Brazil in the last two decades, malaria incidence in gold mining settlements and Indigenous lands is still increasing [3]. Due to the difficulty of timely diagnosis and treatment of miners in Indigenous lands and mining sites, a potential intervention to reduce the mining-driven malaria outbreaks is the ‘Malakit’; a novel effective strategy that relies on self-diagnosis and treatment kits [37,38]. This strategy could contribute to both Brazil’s Malaria National Elimination programme and WHO’s Global Technical Strategy for Malaria targets of reducing malaria cases and deaths by at least 90% in 2030 [1,20,39,40].

### (c) Limitations

It is important to recognize this study has several limitations, including spatial data resolution constraints, underreported and asymptomatic malaria cases, changes in access to healthcare over time, lack of human movement data and the use of a coarse temporal scale (see details in supplementary methods). Furthermore, our analyses rely on strong mechanistic hypotheses for the variables of interest, with mining affecting malaria via multiple causal pathways (electronic supplementary material, figure S1). Consequently, unobserved factors that vary locally across years are not considered in the model and correlated with malaria incidence (e.g. access to healthcare) could confound our estimates or, if interpreted differently, could be more indirect mechanisms underlying the inferred land use–malaria relationships.

## 5. Conclusions

We showed that illegal gold mining is linked to the recent surge in malaria in the Yanomami territory. We estimated that for every 1 s.d. increase in illegal gold mining changes (0.03% cover change), there is an associated 20–46% increase in total malaria incidence 1–2 years later (tables 1 and 2). The increased malaria burden coincided with a surge in illegal gold mining and forest edge habitat and became progressively worse as illegal gold mining increased (figures 2 and 3). These impacts go above and beyond the directional trend in malaria increasing over time across the Yanomami territory—the land use effects appear even after controlling for year and uniquely occurred in areas most affected by land use change—implicating illegal gold mining and the resulting increase in forest edge perimeter as primary drivers. Our research suggests that immediate effects of mining on malaria may be most related to forest edge habitat and mosquito breeding sites, and the longer-term effects might be more related to the establishment of parasites in the population and mercury contamination. Because surges in illegal gold mining, forest edge perimeter and malaria coincided with Bolsonaro’s pro-mining and anti-Indigenous policies, our results highlight the likely influence of his government on the malaria burden suffered by the Yanomami.

## Supplementary Material

S2

S1

S3

Supplementary material is available online [41].

Supplementary material is available online at https://doi.org/10.6084/m9.figshare.c.8154237.

## Figures and Tables

**Table 1.** Two-way fixed-effects panel regression results for total non-lagged and 1 and 2 years lagged mining changes: estimates, standard errors, *z-* and *p*-values of the impact of forest cover, forest growth, forest edge perimeter, mining changes (non-lagged, 1 and 2 years lagged), temperature and precipitation on the incidence of malaria in Yanomami communities. Bold *p*-values indicate statistical difference.

	non-lagged model				1 year lagged model				2 years lagged model			
	estimates	s.e.	z-value	*p*‐value	estimates	s.e.	z-value	*p*‐value	estimates	s.e.	z-value	*p*‐value
forest cover	0.234	0.161	1.453	0.146	0.238	0.161	1.476	0.140	0.230	0.165	1.399	0.162
forest growth	−0.166	0.046	−3.601	**<0.001**	−0.179	0.046	−3.898	**<0.001**	−0.178	0.045	−3.956	**<0.001**
edge perimeter	0.441	0.116	3.912	**<0.001**	0.437	0.113	3.853	**<0.001**	0.428	0.116	3.688	**<0.001**
mining changes	0.005	0.075	0.073	0.942	0.188	0.036	5.268	**<0.001**	0.381	0.063	6.084	**<0.001**
annual temp.	−0.336	0.588	−0.572	0.567	−0.323	0.590	−0.547	0.584	−0.312	0.607	−0.514	0.607
annual prec.	0.064	0.105	0.608	0.543	0.054	0.098	0.547	0.586	0.057	0.105	0.539	0.586

**Table 2.** Two-way fixed-effects panel regression results for total non-lagged and 1 and 2 years lagged mining changes for *P. falciparum* incidence. Estimates, standard errors, *z* and *p* values of the impact of forest cover, forest growth, forest edge perimeter, mining changes (non-lagged, 1 year lagged and 2 years lagged), temperature and precipitation on the incidence of *P. falciparum* infection in Yanomami communities. Bold *p* values indicate statistical difference.

	non-lagged model				1 year lagged model				2 years lagged model			
	estimates	s.e.	z-value	*p*‐value	estimates	s.e.	z-value	*p*‐value	estimates	s.e.	z-value	*p*‐value
forest cover	0.300	0.138	2.173	**0.030**	0.298	0.138	2.153	**0.031**	0.290	0.141	2.065	**0.038**
forest growth	−0.166	0.040	−4.098	**<0.001**	−0.172	0.039	−4.358	**<0.001**	−0.173	0.040	−4.356	**<0.001**
edge perimeter	0.290	0.119	2.441	**0.015**	0.290	0.119	2.434	**0.015**	0.286	0.121	2.357	**0.018**
mining changes	0.033	0.058	0.566	0.571	0.244	0.087	2.806	**0.005**	0.459	0.056	8.227	**<0.001**
annual temp.	−0.102	0.452	−0.225	0.822	−0.109	0.445	−0.245	0.806	−0.099	0.454	−0.219	0.826
*annual prec.*	0.155	0.204	0.761	0.447	0.150	0.196	0.766	0.443	0.163	0.207	0.793	0.428

## Data Availability

Programming codes (GEE and R) and panel data used in this research are available from the Dryad Digital Repository [23] along with the data description. The data used in this research is publicly available and was obtained from the Brazilian Ministry of Health (https://public.tableau.com/app/profile/mal.ria.brasil/vizzes), MapBiomas Brasil (https://brasil.mapbiomas.org), and Climate Research Unit (CRU) database (https://crudata.uea.ac.uk/cru/data/hrg/).
