# Supplementary material for: Investigating the Yanomami malaria outbreak: gold mining and malaria
Source: Biol Lett. Author manuscript; Available in PMC 2026 Mar 12. (PMC12979953; doi:10.1098/rsbl.2025.0659)
Supplement: S2 [file NIHMS2149152-supplement-S2.docx]

**Supplementary Methods: Investigating the Yanomami malaria outbreak: gold mining and malaria**

Daniela de Angeli Dutra, Cor Jesus Fontes, Érika Martins Braga, Erin A. Mordecai

*Land cover variables*

In total, 64 infection sites from 28 polo bases reported malaria cases in Yanomami people (Figures 1 and 2). We used the Google Earth Engine platform to extract annual data on forest (primary and secondary vegetation), illegal gold mining cover, and forest edge perimeter from MapBiomas Brasil Collection 9.0 (https://brasil.mapbiomas.org) within a 10km radius polygon around each infection site from 2003 to 2023. This polygon size was chosen as it captures the surrounding landscape that the Yanomami are likely to use for daily activities (e.g., hunting and agriculture), and it covers an area that is large enough to account for the impacts of mercury contamination from mining sites not located near each infection site. Due to the very high forest cover (>95%), the cover of other land cover variables was not significant (i.e., not captured by MapBiomas Brasil) or presented low to null variation over time and was, therefore, excluded from our analyses. Annual forest growth and illegal gold mining changes were calculated by subtracting current year cover from previous year cover.

*Climatic variables*

Since temperature and precipitation can affect the transmission of malaria, the average annual temperature at 2m from the ground and total annual precipitation were estimated for each site polygon. These climatic variables capture general climate conditions of temperature and precipitation for each site in each year that could influence vector and *Plasmodium* development, such as vector breeding habitat availability and temperature-dependent parasite development. Both temperature and precipitation variables were extracted from the high-resolution (0.5-degree resolution) datasets of the Climate Research Unit (CRU) database (<https://crudata.uea.ac.uk/cru/data/hrg/>). CRU temperature and precipitation values are estimated using interpolation of climate anomalies obtained from weather station observations [1]. To calculate the average annual temperature at 2m for each pixel, monthly values were averaged. For total annual precipitation in each pixel, monthly precipitation values were summed. We then calculated the mean (for temperature) or sum (for precipitation) within each polygon. By estimating annual means for each polygon, we can ensure a consistent and spatially representative dataset that aligns with the annual temporal scale of the analysis of land use effects on malaria. The incorporation of climatic variables in our analysis allows us to control for potential confounding variation that could obscure the causal land use effect estimates of interest. While many other climate indices exist and could be related to malaria incidence and possibly correlated with annual mean temperature and total precipitation, this analysis aims to control for confounding variation, rather than to isolate climate variables that explain the most variation in malaria. All metrics represent the averages for each year separately.

*Spatial autocorrelation checks*

To check if malaria incidence exhibited spatial structure or autocorrelation, we ran Moran’s I test and found very low spatial autocorrelation for malaria incidence in the Yanomami territory (-0.002). From this, we concluded that the analysis did not need to control for confounding variation associated with the spatial structure across infection sites (i.e., infection site-infection site distance).

*Robustness checks*

For robustness checks, we ran five additional models for each different model specification (i.e., total malaria incidence and *P. falciparum* incidence and both lagged and non-lagged mining models). The first model removed climate variables, the second model removed forest cover, the third model removed secondary forest growth, the fourth model removed secondary forest growth and forest edge perimeter, and the fifth model used quadratic temperature to account for possible non-linear effects of temperature. In addition, we repeated the main model setting after removing outlier infection sites (i.e., infection sites where the incidence of malaria reached 2 or more in any given year, N = 7 outlier infection sites. All models were repeated without lagging changes in mining (lag = 0) and lagging mining changes by one and two years (lag = 1 and 2, Sup. Tables 1-12).

The effects of illegal gold mining on malaria were consistent across most lagged model specifications except those removing outliers, both considering all malaria data (Sup. Tables 1-5, 11) and *P. falciparum* only (Sup. Tables 5-10, 12). However, changes in gold mining cover were not associated with malaria incidence in the non-lagged main and robustness check models we ran (Sup Tables 1-12). This suggests that the longer-term impacts of illegal gold mining on malaria incidence are more robust than the short-term effects. One standard deviation of increase in forest growth decreased malaria by 15.3%, this effect was consistent across different lags of gold mining (Table 1) and across robustness checks models (Table 1-2, Sup. Table 1-12). Forest cover was linked to increases in malaria incidence in some models considering both all malaria cases and *P. falciparum* only. However, this effect was inconsistent across main and robustness check models.

*Limitations*

This study has several limitations. First, it relies on land use data that has limited resolution. Satellites may not be able to capture changes in land use on very small scales. In our analyses, the resolution of the satellite imagery is a maximum of 30 meters [2]; therefore, it is possible that some illegal gold mining activities and forest loss are not detected in our analyses because their scale is too small. Moreover, changes in land cover are a proxy that capture both the ecological impacts and the human and social impacts (including mobility). In addition, underreporting of malaria cases in Indigenous populations is a common and known issue, as people in these communities often present a predominance of submicroscopic infection [3]. Robortella et al. (2020) reported that 75-80% of malaria infections in Yanomami communities are submicroscopic [4]. In addition, as the Yanomami are an Indigenous people inhabiting remote areas of the Amazon, they do not always have easy and constant access to healthcare and, as a result, malaria diagnosis. Therefore, it is likely that the number of malaria cases and the malaria incidence reported for the Yanomami are underestimated. In addition, our analyses do not consider changes in access to healthcare over time. If mining was associated with reduced healthcare access, and therefore reduced malaria diagnostic capacity, this would make our estimates of the impact of illegal gold mining on malaria incidence in the Yanomami territory conservative. It is also important to note that our analysis uses an annual temporal scale due to the slow pace of illegal gold mining in most infection sites, not considering seasonal or month-to-month variation in malaria transmission. Instead, we quantified the broad impact of changes in land cover on malaria incidence. Estimates of malaria cases in 2023 only include the months of January to September. Finally, in our analyses, we use a fine spatial resolution (i.e., coordinate points), but it is possible that the impact of illegal gold mining on malaria incidence could be inconsistent at larger geographic scales.

Our analysis relies on strong assumptions about the causal pathways linking land use and land cover changes to variations in malaria incidence. Specifically, it assumes that both unobserved time-invariant and time-varying factors (e.g., changes in land use) have linear effects; if this is violated our estimates could be biased. Moreover, other unobserved year-specific factors that vary across sites (e.g., local changes in healthcare access), also influence malaria incidence, and are not controlled for could also confound our estimates. Due to the absence of human movement data, our models cannot distinguish between the direct environmental impacts of illegal gold mining (e.g., puddle formation) and the effects of miner movement throughout the Yanomami territory when examining non-lagged associations between mining and malaria incidence. Follow-up studies that directly collect information on the mobility of miners and Indigenous people, parasite importation, healthcare access, nutrition, mercury contamination, vector habitat and abundance, and other factors could capture the mining–malaria relationship more mechanistically.

**References**

1. Harris I, Osborn TJ, Jones P, Lister D. 2020 Version 4 of the CRU TS monthly high-resolution gridded multivariate climate dataset. *Sci. Data* **7**, 109. (doi:10.1038/s41597-020-0453-3)

2. Souza CM *et al.* 2020 Reconstructing Three Decades of Land Use and Land Cover Changes in Brazilian Biomes with Landsat Archive and Earth Engine. *Remote Sens.* **12**, 2735. (doi:10.3390/rs12172735)

3. Laporta GZ *et al.* 2025 Intermediate forest cover and malaria risk in an Amazon deforestation frontier. *Acta Trop.* **269**, 107757. (doi:10.1016/j.actatropica.2025.107757)

4. Robortella DR *et al.* 2020 Prospective assessment of malaria infection in a semi-isolated Amazonian indigenous Yanomami community: Transmission heterogeneity and predominance of submicroscopic infection. *PLOS ONE* **15**, e0230643. (doi:10.1371/journal.pone.0230643)
