# Supplementary figures and images for: Investigating the Yanomami malaria outbreak: gold mining and malaria
Source: Biol Lett. Author manuscript; Available in PMC 2026 Mar 12. (PMC12979953; doi:10.1098/rsbl.2025.0659)

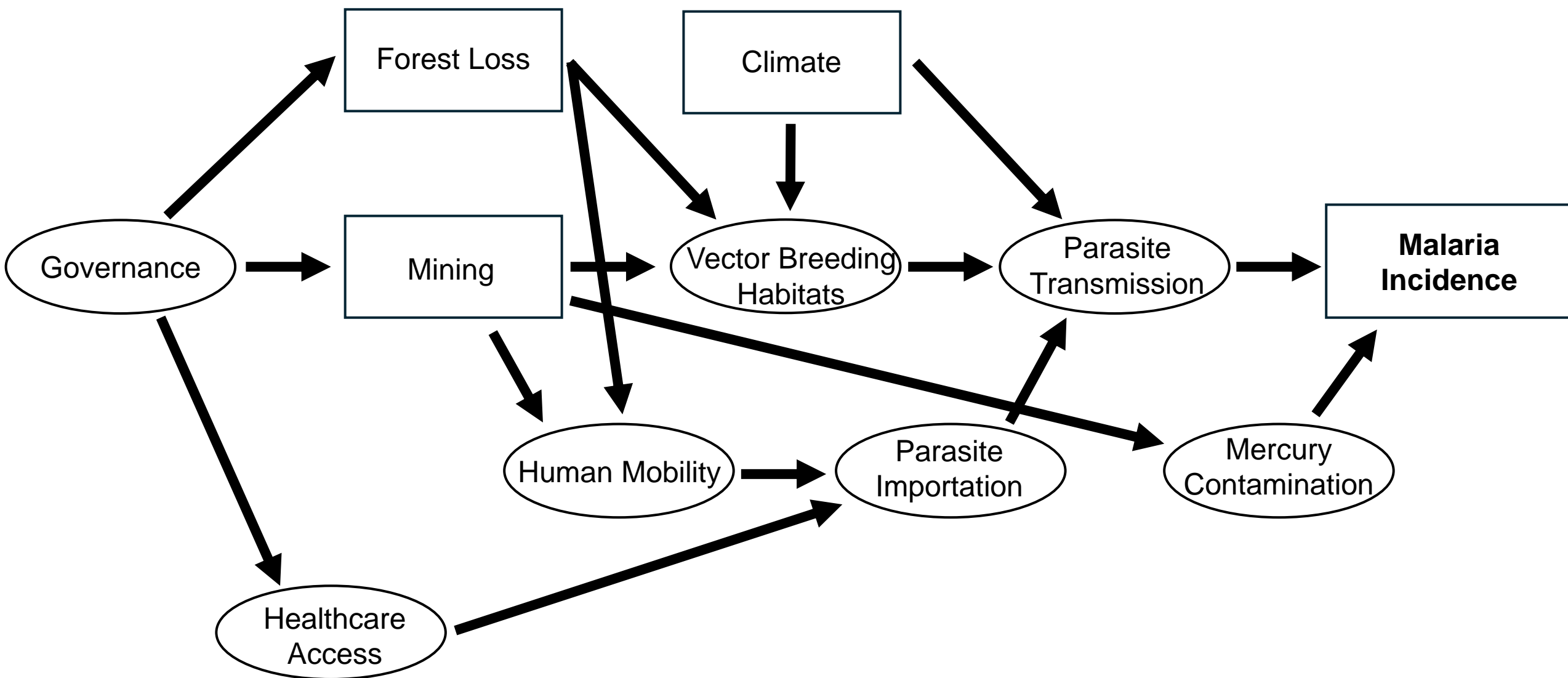

Supplement: S1 [file NIHMS2149152-supplement-S1.pdf]
