# Supplementary material for: Investigating the Yanomami malaria outbreak: gold mining and malaria
Source: Biol Lett. Author manuscript; Available in PMC 2026 Mar 12. (PMC12979953; doi:10.1098/rsbl.2025.0659)
Supplement: S3 [file NIHMS2149152-supplement-S3.docx]

**Supplementary Tables: Investigating the Yanomami malaria outbreak: gold mining and malaria**

Daniela de Angeli Dutra, Cor Jesus Fontes, Érika Martins Braga, Erin A. Mordecai

**Supplementary Table 1: Panel regression robustness analysis dropping climate covariates.** Estimates, standard errors, *z and p* values of the impact of forest cover, forest growth, forest edge perimeter, and mining changes (non-lagged, one-year lagged and two-year lagged) on the total incidence of malaria in Yanomami communities. Bold p-values indicate statistical difference.

|  | non-lagged model | | | | one-year lagged model | | | | two-year lagged model | | | | |
| --- | --- | --- | --- | --- | --- | --- | --- | --- | --- | --- | --- | --- | --- |
|  | Est | S error | z-value | p-value | Est | S error | z-value | p-value | Est | S error | z-value | p-value |  |
| Forest cover | 0.333 | 0.085 | 3.937 | **<0.001** | 0.333 | 0.085 | 3.931 | **<0.001** | 0.322 | 0.085 | 3.802 | **<0.001** |  |
| Forest growth | -0.183 | 0.045 | -4.107 | **<0.001** | -0.195 | 0.045 | -4.374 | **<0.001** | -0.193 | 0.043 | -4.500 | **<0.001** |  |
| Edge perimeter | 0.421 | 0.108 | 3.902 | **<0.001** | 0.419 | 0.109 | 3.834 | **<0.001** | 0.410 | 0.111 | 3.708 | **<0.001** |  |
| Mining change | 0.002 | 0.076 | 0.035 | 0.973 | 0.188 | 0.036 | 5.293 | **<0.001** | 0.381 | 0.064 | 5.963 | **<0.001** |  |

|  | non-lagged model | | | | one-year lagged model | | | | two-year lagged model | | | | |
| --- | --- | --- | --- | --- | --- | --- | --- | --- | --- | --- | --- | --- | --- |
|  | Est | S error | z-value | p-value | Est | S error | z-value | p-value | Est | S error | z-value | p-value |  |
| Forest growth | -0.154 | 0.041 | -3.776 | **<0.001** | -0.166 | 0.040 | -4.149 | **<0.001** | -0.166 | 0.039 | -4.251 | **<0.001** |  |
| Edge perimeter | 0.406 | 0.135 | 3.021 | **0.002** | 0.402 | 0.135 | 2.964 | **<0.001** | 0.395 | 0.139 | 2.845 | **0.005** |  |
| Mining change | 0.007 | 0.073 | 0.101 | 0.920 | 0.187 | 0.037 | 5.096 | **<0.001** | 0.381 | 0.063 | 6.040 | **<0.001** |  |
| Annual temp. | -0.580 | 0.368 | -1.576 | 0.115 | -0.572 | 0.366 | -1.560 | 0.119 | -0.552 | 0.382 | -1.446 | 0.148 |  |
| Annual prec. | 0.009 | 0.101 | 0.869 | 0.385 | 0.078 | 0.093 | 0.0835 | 0.404 | 0.080 | 0.100 | 0.805 | 0.421 |  |

**Supplementary Table 2: Panel regression robustness analysis dropping forest cover.** Estimates, standard errors, *z and p* values of the impact of forest growth, forest edge perimeter, mining changes (non-lagged, one-year lagged and two-year lagged), the average annual temperature at 2m, and total annual precipitation on the total incidence of malaria in Yanomami communities. Bold p-values indicate statistical difference.

|  | non-lagged model | | | | one-year lagged model | | | | two-year lagged model | | | | |
| --- | --- | --- | --- | --- | --- | --- | --- | --- | --- | --- | --- | --- | --- |
|  | Est | S error | z-value | p-value | Est | S error | z-value | p-value | Est | S error | z-value | p-value |  |
| Forest cover | 0.195 | 0.168 | 1.155 | 0.248 | 0.194 | 0.169 | 1.147 | 0.251 | 0.186 | 0.172 | 1.087 | 0.277 |  |
| Edge perimeter | 0.462 | 0.115 | 4.029 | **<0.001** | 0.458 | 0.116 | 3.941 | **<0.001** | 0.452 | 0.119 | 3.802 | **<0.001** |  |
| Mining change | 0.067 | 0.065 | 1.035 | 0.301 | 0.175 | 0.040 | 4.401 | **<0.001** | 0.337 | 0.046 | 7.278 | **<0.001** |  |
| Annual temp. | -0.470 | 0.606 | -0.775 | 0.438 | -0.468 | 0.611 | -0.766 | 0.444 | -0.458 | 0.626 | -0.732 | 0.464 |  |
| Annual prec. | 0.048 | 0.010 | 0.483 | 0.629 | 0.047 | 0.098 | 0.480 | 0.632 | 0.047 | 0.103 | 0.456 | 0.648 |  |

**Supplementary Table 3: Panel regression robustness analysis dropping forest growth.** Estimates, standard errors, *z and p* values of the impact of forest cover, forest edge perimeter, mining changes (non-lagged, one-year lagged and two-year lagged), the average annual temperature at 2m, and total annual precipitation on the total incidence of malaria in Yanomami communities. Bold p-values indicate statistical difference.

|  | non-lagged model | | | | one-year lagged model | | | | two-year lagged model | | | | |
| --- | --- | --- | --- | --- | --- | --- | --- | --- | --- | --- | --- | --- | --- |
|  | Est | S error | z-value | p-value | Est | S error | z-value | p-value | Est | S error | z-value | p-value |  |
| Forest cover | 0.059 | 0.179 | 0.332 | 0.740 | 0.060 | 0.180 | 0.334 | 0.739 | 0.057 | 0.184 | 0.308 | 0.758 |  |
| Mining change | 0.070 | 0.056 | 1.256 | 0.209 | 0.172 | 0.031 | 5.467 | **<0.001** | 0.347 | 0.049 | 7.049 | **<0.001** |  |
| Annual temp. | -0.264 | 0.680 | -0.389 | 0.697 | -0.261 | 0.686 | -0.380 | 0.704 | -0.251 | 0.702 | -0.356 | 0.721 |  |
| Annual prec. | 0.001 | 0.119 | 0.009 | 0.992 | 0.001 | 0.116 | 0.006 | 0.995 | 0.002 | 0.123 | 0.019 | 0.985 |  |

**Supplementary Table 4: Panel regression robustness analysis dropping forest growth and edge perimeter.** Estimates, standard errors, *z and p* values of the impact of forest cover, mining changes (non-lagged, one-year lagged and two-year lagged), the average annual temperature at 2m, and total annual precipitation on the total incidence of malaria in Yanomami communities. Bold p-values indicate statistical difference.

**Supplementary Table 5: Panel regression robustness analysis using quadratic temperature.** Estimates, standard errors, *z and p* values of the impact of forest cover, forest growth, forest edge perimeter, mining changes (non-lagged, one-year lagged and two-year lagged), the average and quadratic annual temperature at 2m, and total annual precipitation on the incidence of malaria in Yanomami communities. Bold p-values indicate statistical difference.

|  | non-lagged model | | | | one-year lagged model | | | | two-year lagged model | | | | |
| --- | --- | --- | --- | --- | --- | --- | --- | --- | --- | --- | --- | --- | --- |
|  | Est | S error | z-value | p-value | Est | S error | z-value | p-value | Est | S error | z-value | p-value |  |
| Forest cover | 0.461 | 0.149 | 3.098 | **0.002** | 0.463 | 0.150 | 3.082 | **0.002** | 0.460 | 0.155 | 2.966 | **0.003** |  |
| Forest growth | -0.137 | 0.037 | -3.724 | **<0.001** | -0.150 | 0.037 | -4.047 | **<0.001** | -0.149 | 0.036 | -4.153 | **<0.001** |  |
| Edge perimeter | 0.370 | 0.096 | 3.856 | **<0.001** | 0.364 | 0.096 | 3.770 | **<0.001** | 0.354 | 0.098 | 3.603 | **<0.001** |  |
| Mining change | 0.011 | 0.071 | 0.156 | 0.876 | 0.175 | 0.037 | 4.754 | **<0.001** | 0.362 | 0.064 | 5.641 | **<0.001** |  |
| Annual temp. | -0.091 | 0.304 | -0.299 | 0.765 | -0.079 | 0.304 | -0.258 | 0.796 | -0.057 | 0.318 | -0.181 | 0.856 |  |
| Quadratic temp | 0.434 | 0.114 | 3.799 | **<0.001** | 0.431 | 0.115 | 3.748 | **<0.001** | 0.438 | 0.118 | 3.712 | **<0.001** |  |
| Annual prec. | 0.205 | 0.126 | 1.626 | 0.104 | 0.196 | 0.121 | 1.620 | 0.105 | 0.205 | 0.124 | 1.620 | 0.105 |  |

|  | non-lagged model | | | | one-year lagged model | | | | two-year lagged model | | | |
| --- | --- | --- | --- | --- | --- | --- | --- | --- | --- | --- | --- | --- |
|  | Est | S error | z-value | p-value | Est | S error | z-value | p-value | Est | S error | z-value | p-value |
| Forest cover | 0.349 | 0.083 | 4.200 | **<0.001** | 0.348 | 0.083 | 4.192 | **<0.001** | 0.339 | 0.083 | 4.073 | **<0.001** |
| Forest growth | -0.172 | 0.033 | -5.177 | **<0.001** | -0.179 | 0.033 | -5.471 | **<0.001** | -0.179 | 0.032 | -5.595 | **<0.001** |
| Edge perimeter | 0.277 | 0.116 | 2.382 | **0.017** | 0.277 | 0.118 | 2.340 | **0.020** | 0.273 | 0.120 | 2.271 | **0.023** |
| Mining change | 0.044 | 0.051 | 0.875 | 0.382 | 0.243 | 0.087 | 2.796 | **0.005** | 0.453 | 0.056 | 8.029 | **<0.001** |

**Supplementary Table 6: Panel regression robustness analysis dropping climate covariates for *P. falciparum* incidence.** Estimates, standard errors, *z and p* values of the impact of forest cover, forest growth, forest edge perimeter, and mining changes (non-lagged, one-year lagged and two-year lagged) on the incidence of *P. falciparum* in Yanomami communities. Bold p-values indicate statistical difference.

|  | non-lagged model | | | | one-year lagged model | | | | two-year lagged model | | | | |
| --- | --- | --- | --- | --- | --- | --- | --- | --- | --- | --- | --- | --- | --- |
|  | Est | S error | z-value | p-value | Est | S error | z-value | p-value | Est | S error | z-value | p-value |  |
| Forest growth | -0.150 | 0.034 | -4.426 | **<0.001** | -0.155 | 0.032 | -4.801 | **<0.001** | -0.157 | 0.032 | -4.834 | **<0.001** |  |
| Edge perimeter | 0.238 | 0.133 | 1.794 | 0.073 | 0.238 | 0.132 | 1.807 | 0.071 | 0.236 | 0.133 | 1.770 | 0.077 |  |
| Mining change | 0.031 | 0.055 | 0.569 | 0.569 | 0.248 | 0.091 | 2.731 | **0.006** | 0.465 | 0.064 | 7.195 | **<0.001** |  |
| Annual temp. | -0.453 | 0.280 | -1.620 | 0.105 | -0.458 | 0.270 | -1.700 | 0.089 | -0.441 | 0.278 | -1.588 | 0.112 |  |
| Annual prec. | 0.185 | 0.200 | 0.925 | 0.355 | 0.178 | 0.192 | 0.929 | 0.353 | 0.192 | 0.203 | 0.949 | 0.343 |  |

**Supplementary Table 7: Panel regression robustness analysis dropping forest cover for *P. falciparum* incidence.** Estimates, standard errors, *z and p* values of the impact of forest growth, forest edge perimeter, mining changes (non-lagged, one-year lagged and two-year lagged), the average annual temperature at 2m, and total annual precipitation on the incidence of *P. falciparum* in Yanomami communities. Bold p-values indicate statistical difference.

**Supplementary Table 8: Panel regression robustness analysis dropping forest growth for *P. falciparum* incidence.** Estimates, standard errors, *z and p* values of the impact of forest cover, forest edge perimeter, mining changes (non-lagged, one-year lagged and two-year lagged), the average annual temperature at 2m, and total annual precipitation on the total incidence of malaria in Yanomami communities. Bold p-values indicate statistical difference.

|  | non-lagged model | | | | one-year lagged model | | | | two-year lagged model | | | |
| --- | --- | --- | --- | --- | --- | --- | --- | --- | --- | --- | --- | --- |
|  | Est | S error | z-value | p-value | Est | S error | z-value | p-value | Est | S error | z-value | p-value |
| Forest cover | 0.249 | 0.160 | 1.553 | 0.120 | 0.242 | 0.160 | 1.506 | 0.132 | 0.234 | 0.163 | 1.434 | 0.151 |
| Edge perimeter | 0.311 | 0.117 | 2.664 | **0.007** | 0.231 | 0.078 | 2.943 | **0.003** | 0.308 | 0.119 | 2.594 | **0.009** |
| Mining change | 0.088 | 0.060 | 1.452 | 0.147 | 0.311 | 0.118 | 2.641 | **0.008** | 0.417 | 0.043 | 9.759 | **<0.001** |
| Annual temp. | -0.266 | 0.493 | -0.534 | 0.589 | -0.280 | 0.491 | -0.570 | 0.568 | -0.276 | 0.497 | -0.554 | 0.579 |
| Annual prec. | 0.145 | 0.200 | 0.727 | 0.467 | 0.153 | 0.196 | 0.780 | 0.435 | 0.162 | 0.204 | 0.791 | 0.428 |

**Supplementary Table 9: Panel regression robustness analysis dropping forest growth and forest edge perimeter for *P. falciparum* incidence.** Estimates, standard errors, *z and p* values of the impact of forest cover, mining changes (non-lagged, one-year lagged and two-year lagged), the average annual temperature at 2m, and total annual precipitation on the total incidence of malaria in Yanomami communities. Bold p-values indicate statistical difference.

|  | non-lagged model | | | | one-year lagged model | | | | two-year lagged model | | | |
| --- | --- | --- | --- | --- | --- | --- | --- | --- | --- | --- | --- | --- |
|  | Est | S error | z-value | p-value | Est | S error | z-value | p-value | Est | S error | z-value | p-value |
| Forest cover | 0.142 | 0.156 | 0.910 | 0.363 | 0.135 | 0.116 | 0.866 | 0.387 | 0.129 | 0.159 | 0.814 | 0.416 |
| Mining change | 0.081 | 0.056 | 1.451 | 0.147 | 0.232 | 0.078 | 2.982 | **0.003** | 0.425 | 0.033 | 12.894 | **<0.001** |
| Annual temp. | -0.132 | 0.476 | -0.278 | 0.781 | -0.145 | 0.478 | -0.304 | 0.760 | -0.140 | 0.484 | -0.290 | 0.772 |
| Annual prec. | 0.114 | 0.209 | 0.547 | 0.584 | 0.120 | 0.203 | 0.589 | 0.556 | 0.130 | 0.212 | 0.614 | 0.539 |

|  | non-lagged model | | | | one-year lagged model | | | | two-year lagged model | | | | |
| --- | --- | --- | --- | --- | --- | --- | --- | --- | --- | --- | --- | --- | --- |
|  | Est | S error | z-value | p-value | Est | S error | z-value | p-value | Est | S error | z-value | p-value |  |
| Forest cover | 0.460 | 0.131 | 3.500 | **<0.001** | 0.453 | 0.133 | 3.412 | **<0.001** | 0.445 | 0.136 | 3.278 | **0.001** |  |
| Forest growth | -0.141 | 0.030 | -4.696 | **<0.001** | -0.147 | 0.029 | -4.980 | **<0.001** | -0.148 | 0.029 | -5.030 | **<0.001** |  |
| Edge perimeter | 0.249 | 0.112 | 2.214 | **0.027** | 0.247 | 0.113 | 2.182 | **0.029** | 0.244 | 0.115 | 2.116 | **0.034** |  |
| Mining change | 0.032 | 0.056 | 0.588 | 0.557 | 0.237 | 0.090 | 2.638 | **0.008** | 0.449 | 0.059 | 7.563 | **<0.001** |  |
| Annual temp. | -0.006 | 0.212 | -0.028 | 0.977 | -0.014 | 0.206 | -0.069 | 0.945 | -0.004 | 0.216 | -0.018 | 0.986 |  |
| Quadratic temp | 0.368 | 0.110 | 3.354 | **<0.001** | 0.362 | 0.113 | 3.212 | **0.001** | 0.359 | 0.114 | 3.155 | **0.002** |  |
| Annual prec. | 0.280 | 0.202 | 1.386 | 0.166 | 0.273 | 0.197 | 1.387 | 0.165 | 0.286 | 0.206 | 1.388 | 0.165 |  |

**Supplementary Table 10: Panel regression robustness analysis using quadratic temperature for *P. falciparum* incidence.** Estimates, standard errors, *z and p* values of the impact of forest cover, forest growth, forest edge perimeter, mining changes (non-lagged, one-year lagged and two-year lagged), the average and quadratic annual temperature at 2m, and total annual precipitation on the incidence of malaria in Yanomami communities. Bold p-values indicate statistical difference.

|  | non-lagged model | | | | one-year lagged model | | | | | two-year lagged model | | | | |
| --- | --- | --- | --- | --- | --- | --- | --- | --- | --- | --- | --- | --- | --- | --- |
|  | Est | S error | z-value | p-value | Est | S error | z-value | p-value | Est | | S error | z-value | p-value |  |
| Forest cover | 0.243 | 0.165 | 1.476 | 0.140 | 0.243 | 0.165 | 1.473 | 0.141 | 0.237 | | 0.167 | 1.414 | 0.157 |  |
| Forest growth | -0.180 | 0.056 | -3.221 | **<0.001** | -0.184 | 0.056 | -3.249 | **0.016** | -0.186 | | 0.057 | -3.254 | **0.001** |  |
| Edge perimeter | 0.431 | 0.111 | 3.900 | **<0.001** | 0.422 | 0.114 | 3.715 | **<0.001** | 0.412 | | 0.116 | 3.548 | **<0.001** |  |
| Mining change | -0.104 | 0.063 | -1.655 | 0.098 | 0.159 | 0.032 | 4.942 | **<0.001** | 0.758 | | 0.490 | 1.545 | 0.122 |  |
| Annual temp. | -0.326 | 0.602 | -0.542 | 0.588 | -0.315 | 0.610 | -0.517 | 0.605 | -0.305 | | 0.626 | -0.487 | 0.626 |  |
| Annual prec. | -0.001 | 0.073 | -0.019 | 0.984 | -0.007 | 0.069 | -0.105 | 0.916 | -0.017 | | 0.069 | -0.249 | 0.804 |  |

**Supplementary Table 11: Panel regression robustness analysis removing outliers (i.e., infection sites where malaria incidence was above 2).** Estimates, standard errors, *z and p* values of the impact of forest cover, forest growth, forest edge perimeter, mining changes (non-lagged, one-year lagged and two-year lagged), the average annual temperature at 2m, and total annual precipitation on the incidence of malaria in Yanomami communities. Bold p-values indicate statistical difference.

|  | non-lagged model | | | | one-year lagged model | | | | two-year lagged model | | | | |
| --- | --- | --- | --- | --- | --- | --- | --- | --- | --- | --- | --- | --- | --- |
|  | Est | S error | z-value | p-value | Est | S error | z-value | p-value | Est | S error | z-value | p-value |  |
| Forest cover | 0.314 | 0.142 | 2.215 | **0.027** | 0.312 | 0.142 | 2.193 | **0.028** | 0.306 | 0.145 | 2.116 | **0.034** |  |
| Forest growth | -0.183 | 0.048 | -3.836 | **<0.001** | -0.183 | 0.048 | -3.777 | **<0.001** | -0.183 | 0.049 | -3.757 | **<0.001** |  |
| Edge perimeter | 0.274 | 0.126 | 2.180 | **0.029** | 0.273 | 0.127 | 2.158 | **0.031** | 0.271 | 0.127 | 2.127 | **0.033** |  |
| Mining change | -0.037 | 0.021 | -1.743 | 0.081 | 0.070 | 0.884 | 0.794 | 0.427 | 0.380 | 0.412 | 0.921 | 0.357 |  |
| Annual temp. | -0.034 | 0.457 | -0.074 | 0.941 | -0.031 | 0.460 | -0.067 | 0.947 | -0.028 | 0.465 | -0.061 | 0.952 |  |
| Annual prec. | 0.163 | 0.192 | 0.850 | 0.396 | 0.163 | 0.192 | 0.849 | 0.396 | 0.158 | 0.193 | 0.820 | 0.412 |  |

**Supplementary Table 12: Panel regression robustness analysis removing outliers for *P. falciparum* incidence.** Estimates, standard errors, *z and p* values of the impact of forest cover, forest growth, forest edge perimeter, mining changes (non-lagged, one-year lagged and two-year lagged), the average annual temperature at 2m, and total annual precipitation on the incidence of malaria in Yanomami communities. Bold p-values indicate statistical difference.
